# Genomic landscape of non‐small‐cell lung cancer with methylthioadenosine phosphorylase (MTAP) deficiency

**DOI:** 10.1002/cam4.4971

**Published:** 2022-06-23

**Authors:** Prashanth Ashok Kumar, Stephen L. Graziano, Natalie Danziger, Dean Pavlick, Eric A. Severson, Shakti H. Ramkissoon, Richard S. P. Huang, Brennan Decker, Jeffrey S. Ross

**Affiliations:** ^1^ Upstate Cancer Center Upstate Medical University Syracuse New York USA; ^2^ Foundation Medicine Cambridge Massachusetts USA

**Keywords:** comprehensive genomic profiling, methylthioadenosine phosphorylase (MTAP), non‐small‐cell lung cancer, protein arginine methyl transferase, synthetic lethality

## Abstract

**Introduction:**

New treatment strategies for advanced non‐small‐cell lung carcinoma (NSCLC) include synthetic lethality targets focused on protein arginine methyl transferases such as *PRMT5* that exploit the impact of genomic loss of methylthioadenosine phosphorylase (*MTAP)*.

**Methods:**

Twenty nine thousand three hundred seventy nine advanced NSCLC cases underwent hybrid‐capture based comprehensive genomic profiling between June 1, 2018 and May 31, 2020. PD‐L1 expression was determined by immunohistochemistry (Dako 22C3 PharmDx assay).

**Results:**

13.4% (3928/29,379) NSCLC cases exhibited *MTAP* loss distributed in adenocarcinoma (59%), squamous cell carcinoma (22%), NSCLC not otherwise specified (16%), and 1% each for large‐cell neuroendocrine, sarcomatoid, and adenosquamous carcinoma. Statistically significant differences in mitogenic driver alterations included more *KRAS* G12C mutations in *MTAP*‐intact versus *MTAP‐*lost (12% vs. 10%, *p* = 0.0003) and fewer *EGFR* short variant mutations in *MTAP*‐intact versus *MTAP*‐lost NSCLC (10% vs. 13%, *p* < 0.0001). Statistically significant differences in currently untargetable genomic alterations included higher frequencies of *TP53* (70% vs. 63%, *p* < 0.0001) and *RB1* inactivation (10% vs. 2%, *p* < 0.0001) in *MTAP*‐intact compared to *MTAP*‐lost NSCLC. *SMARCA4* inactivation (7% vs. 10%, *p* < 0.0001) was less frequent in *MTAP*‐intact versus *MTAP*‐lost NSCLC. Alterations in *ERBB2*, *MET*, *ALK, ROS1*, and *NTRK1* did not significantly differ between the two groups. Predictors of immunotherapy efficacy were higher in *MTAP*‐intact versus *MTAP*‐lost NSCLC including tumor mutational burden (9.4 vs. 8.6 mut/Mb, *p* = 0.001) and low (30% vs. 28%, *p* = 0.01) and high PD‐L1 (32% vs. 30%, *p* = 0.01) expression. Alterations in biomarkers potentially predictive of immune checkpoint inhibitor resistance (*STK11*, *KEAP1*, and *MDM2*) were similar in the two groups.

**Conclusions:**

*MTAP* loss occurs in 13% of NSCLC, supporting the development of targeted therapies to exploit PRMT5 hyper‐dependence. *MTAP* loss is accompanied by small differences in targeted and immunotherapy options which may impact future combination strategies.

## Graphical abstract

MTAP loss occurs in 13% of non‐small‐cell lung cancers, raising the possibility of targeted treatment with synthetic lethality agents such as PRMT5 inhibitors.

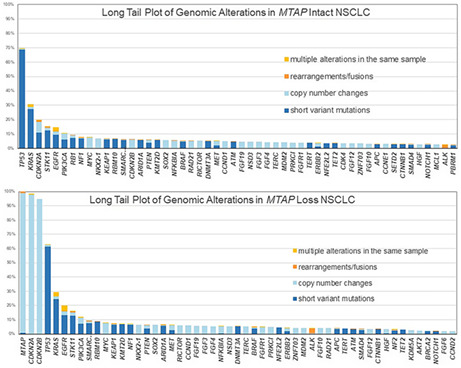

## 1 INTRODUCTION

Despite advances in lung cancer care, many patients are still diagnosed at an advanced stage and have unfavorable outcomes.^1^ Historical 5‐year survival for patients with metastatic non‐small‐cell lung cancer (NSCLC) is approximately 5.5% indicating the need for research enabling more precise utilization of targeted therapies.^2^, ^3^ While predictive biomarkers and novel therapeutic agents have improved survival and quality of life, the extent of this benefit is still undefined.^1^, ^2^, ^4^ Lung cancers with *EGFR* and *ALK* alterations are now treatable with tyrosine kinase inhibitors (TKIs).^5^ Programmed death ligand‐1 (PD‐L1) is a therapeutic target, with immune checkpoint inhibitors (ICPIs) such as pembrolizumab and nivolumab currently being utilized for pretreated metastatic NSCLC and more recently in neoadjuvant protocols.^6^ Overexpression of protein arginine methyltransferase 5 (PRMT5) in malignant lung cells has to lead to the development of inhibitory molecules like GSK3326595,^7^ PRT811,^8^ and JNJ‐64619178,^9^ currently under investigation in advanced solid malignancies including NSCLC.^10^

Methylthioadenosine phosphorylase (MTAP) is a key enzyme in the methionine salvage pathway, responsible for regenerating methionine and adenine.^11^ Based on principles of synthetic lethality, as *MTAP* becomes deficient in a tumor cell, its substrate methylthioadenosine (MTA) will build up.^12^ *MTA* in turn inhibits *PRMT5*, supporting the hypothesis that MTAP deficient lung cancer may respond better to *PRMT5* inhibition.^13^, ^14^ Chromosome 9p21 encodes the *MTAP* gene surrounded by the *miR‐31* and *CDKN2A* genes. In most tumors, *MTAP* is co‐deleted with *CDKN2A* and *CDKN2B* which encode the tumor suppressors p14arf, p16INK4a, and p15Ink4b.^14^ NSCLC patients with 9p21 deletions have shown poorer overall and disease‐free survivals. *MTAP* and *CDKN2A* may function as complex coregulators and a better understanding of its role in cancers like NSCLC may have important clinical implications.^10^ While preclinical models overwhelmingly support the occurrence of cell death potentiated by *MTAP* loss resulting from *PRMT5* inhibition in malignant lung cells that overexpress them, clinical data on human specimens are limited in published literature.^15^

Given the potential of *PRMT5* inhibition in NSCLC and the above‐described interconnection between *PRTM5, MTAP,* and *CDKN2A*,^10^ we examined the genomic alterations occurring in *MTAP‐*deleted NSCLC cases and assessed the clinical implications of *MTAP* loss on established predictive and prognostic biomarkers.

## 2 METHODS

### 2.1 Sample and setting

DNA was extracted from 29,379 clinically advanced NSCLC specimens. Using the submitted clinical and pathology reports, tissue samples were classified as either obtained from metastatic site biopsies or from sites of unresectable loco‐regional disease. The study was approved by the Western Institutional Review Board (Protocol No. 20152817) which issued an approval waiver of informed consent and a HIPAA waiver of authorization.

### 2.2 Analysis

The central laboratory (Foundation Medicine) used for comprehensive genomic profiling (CGP) is Clinical Laboratory Improvement Amendments (CLIA)‐certified and accredited by the College of American Pathologists. A minimum of 50 ng of DNA was extracted from cases that had a minimum of 20% tumor nuclei. After library preparation, adaptor‐ligation based hybrid capture was performed for all coding exons from 287 (version 1) to 324 (version 3) cancer‐related genes plus select introns from 19 (version 1) to 28 (version 3) genes frequently rearranged in cancer. The Illumina HiSeq was used for DNA sequencing to a mean exon coverage depth of >500X. Microsatellite instability (MSI) status was determined on 95 loci. Tumor mutational burden (TMB) was determined using 0.9–1.1 Mb of sequenced DNA. The DAKO 22C3 CDx assay was used to determine PD‐L1 expression using 5‐micron tissue sections. Following the CDx assay guidelines a tumor proportion score (TPS) was determined for each sample stained with the DAKO 22C2 CDx assay. TPS = (positive tumor cells/total tumor cell) × 100. TPS of 0% was defined as negative, low‐level staining defined as 1%–49% TPS, and high‐level staining defined as ≥50% TPS.^16^, ^17^, ^18^, ^19^, ^20^

### 2.3 Statistical analyses

Differences in sample medians were assessed using the unpaired Mann–Whitney–Wilcoxon test. Differences among categorical variables were assessed using chi square test with Yates correction. Statistical tests were 2‐sided and used a significance threshold of *p* < 0.05. Reported *p* values were not adjusted for multiple testing.

## 3 RESULTS

From a cohort of varied solid tumors sequenced for MTAP loss, 29,379/177,705 (16.5%) were NSCLC. All patients had clinically advanced disease with the preponderance of patients having Stage IV disease at the time of sequencing. The clinical and genomic findings in the NSCLC cases are shown in Table 1. 3928/29,379 (13.4%) of all NSCLC cases exhibited *MTAP* loss. The MTAP deleted NSCLC cohort represented the largest subset of tumor type with *MTAP* loss in all sequenced solid tumors. The overall frequency of *MTAP* loss in all tumor types was 9.4%. In the *MTAP*‐lost NSCLC cohort, 59% were adenocarcinomas (LUAD) (12% *MTAP* loss), 22% were squamous cell carcinomas (LUSC) (14% *MTAP* loss), 16% were NSCLC not otherwise specified (NOS) with genomic profiles most consistent with LUAD (14% *MTAP* loss), 1% were large cell neuroendocrine carcinomas (LNEC) (9% *MTAP* loss), 1% were sarcomatoid carcinomas (SRC) (23% *MTAP* loss), and 1% were adenosquamous carcinomas (LUAS) (21% *MTAP* loss). The gender and patient age distributions among the different groups were not significantly different.

**TABLE 1:** Clinical and genomic findings in MTAP intact and MTAP loss NSCLC

	NSCLC *MTAP* Intact	NSCLC *MTAP* loss	*p*‐value
Number of cases	25,843	3928	
Males/Females	50%/50%	50%/50%	NS
Median age (range) years	68 (12–89+)	69 (18–89+)	NS
Currently untargetable genomic alterations			
*CDKN2A*	20%	98%	<0.0001
*CDKN2B*	6%	95%	<0.0001
*TP53*	70%	63%	<0.0001
*KRAS* (all)	31%	29%	NS
*RB1*	10%	2%	<0.0001
*SMARCA4*	7%	10%	<0.0001
Currently targetable genomic alterations in NSCLC			
*KRAS* (G12C)	12%	10%	=0.0003
*EGFR SV only*	10%	13%	<0.0001
*ALK*	3%	4%	NS
*ROS1*	1%	1%	NS
*NTRK1*	1%	1%	NS
*MET*	5% (3% amp)	6% (3% amp)	NS
Currently targetable genomic alterations in non‐NSCLC tumor types			
*ERBB2*	4% (2%amp)	4% (2% amp)	NS
*BRAF*	5%	5%	NS
MTOR pathway targets			
*PIK3CA*	11%	12%	NS
*PTEN*	6%	6%	NS
*NF1*	8%	7%	NS
Immunotherapy response linked genomic alterations			
*STK11*	15%	16%	NS
*KEAP1*	7%	7%	NS
*MDM2*	4%	4%	NS
Immunotherapy response linked biomarkers			
MSI High	0.4%	0.2%	NS
Median TMB	6.3	6.3	NS
Mean TMB	9.4	8.6	=0.001
TMB ≥ 10 mut/Mb	35%	32%	=0.0002
TMB ≥ 20 mut/Mb	10%	8%	<0.0001
PD‐L1 Low Positive	30% (13,931)	28% (2125)	=0.01
PD‐L1 High Positive	32%	30%	=0.01

Abbreviations: NSCLC, non‐small cell lung cancer; MTAP, methylthioadenosine phosphorylase; TMB, tumor mutational burden; MSI, microsatellite instability; mut, mutations; Mb, megabase.

**FIGURE 1:**
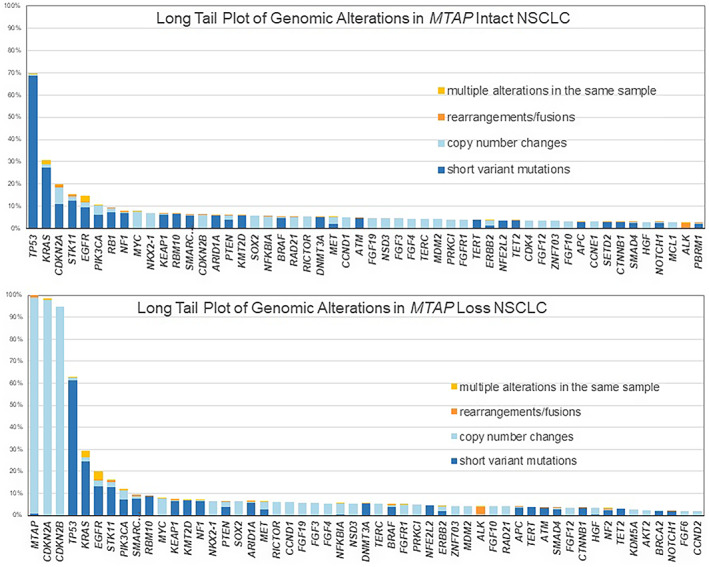
Genomic alterations in *MTAP* intact and *MTAP* loss non‐small cell lung cancer

The distribution of genomic alterations in the *MTAP*‐intact and *MTAP*‐lost NSCLC are shown in Figure 1. The frequencies of co‐deletion (loss) in the *MTAP* loss NSCLC were 98% for *CDKN2A* compared to 20% in *MTAP* intact NSCLC (*p* < 0.0001) and 95% for *CDKN2B* in MTAP loss compared to 6% *MTAP* intact NSCLC (*p* < 0.0001)]. Among currently untargetable genomic alterations, there were higher frequencies of *TP53* (70% vs. 63%, *p* < 0.0001) and *RB1* inactivation (10% vs. 2%, *p* < 0.0001) in the *MTAP*‐intact NSCLC cohort and a higher frequency of *SMARCA4* inactivation (7 vs. 10%, *p* < 0.0001) in the *MTAP*‐loss NSCLC cohort. The non‐G12C *KRAS* genomic alteration frequencies did not differ significantly in the two groups.

There were also significant differences in the frequencies of currently targetable genomic alterations related to FDA‐approved targeted therapies in NSCLC that included a slightly higher frequency of *KRAS* G12C in *MTAP*‐intact NSCLC (12% vs. 10%, *p* = 0.003) and slightly higher frequency of *EGFR* short variant mutations in *MTAP*‐lost NSCLC (10% vs. 13%, *p* < 0.001). The frequencies of alterations in *ERBB2*, *BRAF*, *MET*, *ALK, ROS1,* and *NTRK1* were similar in both *MTAP*‐intact and *MTAP*‐loss NSCLC. Genomic alterations linked to resistance to ICPI‐based treatments including *STK11*, *KEAP1*, and *MDM2* were similar in both groups. Biomarkers linked potential ICPI efficacy were higher in *MTAP‐*intact NSCLC including higher TMB (9.4 vs. 8.6, *p* = 0.001) and low (30% vs. 28%, *p* = 0.01) and high (32% vs. 30%, *p* = 0.01) PD‐L1 IHC staining.

**FIGURE 2:**
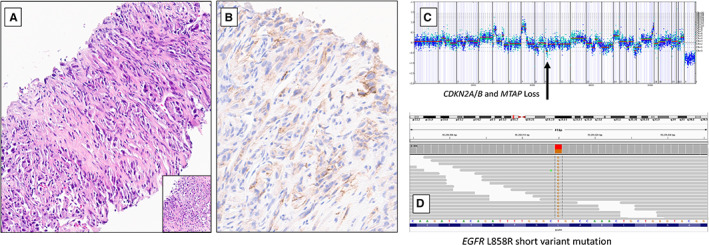
Clinically advanced NSCLC with sarcomatoid features in a 64‐year‐old man. Hematoxylin and eosin‐stained image of the tumor at low magnification (4X) and high magnification in the inset (20X) are shown (A). This tumor stained positively for TTF‐1 and Napsin A. PD‐L1 tumor cell expression was 40% using the DAKO 22C3 assay (B). On comprehensive genomic profiling, the tumor had co‐deletion of *CDKN2A*, *CDKN2B*, and *MTAP*, and was microsatellite stable and TMB‐Low (1 mutation/Mb of sequenced DNA) (C). There was an activating *EGFR* L858R mutation (D). Potential therapy options for this patient would include both anti‐*EGFR* targeted therapies and, despite the low TMB, immunotherapy given the strong anti‐PD‐L1 IHC staining

An example of a NSCLC with sarcomatoid feature exhibiting loss of *MTAP*, *CDKN2A*, and *CDKN2B* is shown in Figure 2. This patient's tumor exhibited 40% PD‐L1 expression and an activating L858R short variant mutation in *EGFR*.

## 4 DISCUSSION

This retrospective cohort study of 29,379 NSCLC cases undergoing CGP revealed *MTAP* loss in 13.4% of cases. Estimates of the frequency of *MTAP* loss in patients with NSCLC previously published in literature ranged from 29% to 61.2% using IHC and 10.1% to 38% using PCR. Prior studies had a relatively smaller sample size ranging from 50 to 165.^21^, ^22^, ^23^, ^24^ According to the AACR GENIE portal, 5.7% (126/2229) non‐small‐cell lung cancer samples exhibited two‐copy deletion of *MTAP* on 9p21.3.^25^, ^26^ Whereas in the COSMIC database, 14.8% (149/1006) of all lung cancers exhibited the deletion.^27^, ^28^A comparison of the studies analyzing *MTAP* loss in NSCLC cases is shown in Table 2. *MTAP* loss is relatively frequent in NSCLC and since it might portend better outcomes with *PRMT5* inhibitors, represents a potential biomarker for treatment response.

**TABLE 2:** Comparison of studies analyzing MTAP loss in NSCLC specimens

S. no	Study	All NSCLC *MTAP* Loss in % (NSCLC with MTAP loss [total NSCLC samples analyzed])	NSCLC subtypes with *MTAP* loss in %	Other findings
1	Current study [CGP]	13.4% (3928/29,379)	LUAD 12% LUSC 14% NSCLC NOS 14% LNEC 9% SRC 23% LUAS 21%	See results section
2	Schmid et al.^22^ [PCR]	38% (19/50)	LUAD 44% LUSC 29% LCLC 50%	homozygous p16INK4A exon 1 alpha in 18% [9(50)]
3	Jing et al.^21^ [Low MTAP expression by IHC]	61.2% (101/165)	All were LUAD	No statistically significant difference in EGFR status between MTAP low and high Median PFS and survival time in the MTAP low was lower (8.1 vs 13.1 months, p = 0.002 and 22 vs. 32 months, p = 0.044).
4	Su et al.^23^ [Low MTAP expression by IHC]	38.4% (38/99)	LUAD 29% LUSC 51.6% LCLC 66.7% P = 0.037 compared to MTAP high	24.2% [24(99)] had both MTAP and p16 loss. This group had poor prognosis compared to those with high expression of both.
5	Watanabe et al.^24^ [IHC and PCR]	*IHC* 28.98% (20/69) *Real Time PCR* 10.14% (7/69)	*IHC* LUAD 15.79% LUSC 46.15% LCLC 100% *Real time PCR* LUAD 5.26% LUSC19.23%	
6	cBioPortal from the AACR Project GENIE^25^, ^26^	5.7% (126/2229)	NSCLC	
7	COSMIC^27^, ^28^	14.81% (149/1006)	All lung cancers	

Abbreviations: LUAD, adenocarcinoma; NSCLC, non small cell lung cancer; NOS, not otherwise specified; LUSC, squamous cell carcinoma;LCLC, large cell lung carcinoma; LNEC, large cell neuroendocrine carcinoma; SRC, sarcomatoid carcinoma; LUAS, adenosquamous carcinoma; CGP, comprehensive genomic profiling; IHC, immunohistochemistry; PCR, polymerase chain reaction; MTAP, methylthioadenosine phosphorylase; GA, genomic alterations.

Through histone methylation of the miR‐99 family and activation of Erk1/2 and Akt pathway, *PRMT5* has been implicated in the progression of lung cancers.^29^ *PRMT5* is an inhibitor of tumor suppressor genes and induces methylation of p53, disrupting its ability to cause death of malignant cells. *PRMT5* also promotes cyclin kinase‐dependent neoplastic growth. The clinical paradigm of *MTAP*‐deficient cells, by building up MTA, which is a potent inhibitor of *PRTM5*, was studied as early as 1981. A number of phase 2 clinical trials attempted to treat *MTAP*‐low tumors including NSCLC and mesothelioma with continuous infusion of L‐alanosine, but failed to reveal clinically significant results.^14^ Experiments with human lung cancer cells lines have revealed that *PRMT5* inhibitor molecules promoted lung cancer cell apoptosis and increased chemosensitivity.^15^ The *PRMT5* inhibitor arginine methyltransferase inhibitor 1 (AMI‐1) caused cell‐cycle arrest in lung adenocarcinoma cells, which was more pronounced when combined with cisplatin.^29^ Yong et al. studied the effect of GSK591, a *PRMT5* inhibitor on lung cancer cell lines. They showed that *PRMT5* inhibition reduced Akt/GSK3β phosphorylation and cyclin D1 and E1 expression, enhancing the apoptosis effect and chemosensitivity caused by resveratrol.^15^ The type 1 *PRMT* inhibitor, GSK3368715 was analyzed in 249 cancer cell lines representing 12 different tumors and was shown to have significant tumor‐suppressive activity, that synergizes on the addition of a *PRMT5* inhibitor (GSK3368715). It was also shown that *MTAP* loss increased the sensitivity of the cell lines to the action of these inhibitors, highlighting its therapeutic potential.^13^

Jing et al. retrospectively reviewed 165 advanced NSCLC treated with platinum‐based chemotherapy and bevacizumab. IHC was used to identify *MTAP*‐low patients, who when compared to *MTAP*‐high patients, had lower progression‐free survival (PFS). This study showed that *MTAP* low status negatively impacted survival in advanced LUAD (HR 1.36, *p* = 0.038).^21^ Other evolving targets of interest are the methionine adenosyltransferase, *MAT1a* and *MAT2a*. They are important cofactors in the polyamine biosynthesis cycle and play an essential role in in the growth and survival of cells. In vivo models have shown that *MAT2a* knockdown reduced the growth and development of *MTAP* deficient tumor cells.^30^ IDE397, a small molecule inhibitor of *MAT2A*, is under investigation as a part of a Phase I trial for advanced solid tumors with *MTAP* deletion.^31^ This proves that prospective clinical trials on *MTAP* deficient NSCLC are greatly needed.

In our study, NSCLC with *MTAP* loss, as expected, was almost always accompanied by the deletion of C*DKN2A* and *CDKN2B*. Thus, it may be beneficial if future studies and trials analyze the utility of CDK inhibitors in *MTAP* deficient NSCLC. *CDKN2A* deletion, promoter methylation, and other alterations cause inactivation of the tumor suppressor p16.^32^ Homozygous deletion of *CDKN2A*/*p16* may cause LUAD to have a poor response to anti‐*EGFR* TKIs. A study of 127 advanced LUAD showed that in *EGFR* mutated patients treated with anti‐*EGFR* TKIs, the median PFS was significantly lower in patients with *CDKN2A*/*p16* deletion compared to those without it (5.3 vs. 10.5 months, *p* = 0.001).^33^ CDK inhibitors have been evaluated in several clinical trials but with limited success and no real clinical utility thus far. The phase III JUNIPER trail in *KRAS‐*mutant Stage IV NSCLC showed that median PFS, response rate, and disease control rate were better with abemaciclib, a third‐generation CDK inhibitor compared to erlotinib. However, the latter had very low efficacy to begin with, thus questioning the utility of the results. Several phase I and II clinical trials are actively investigating third‐generation CDK inhibitors in NSCLC, including palbociclib, ribociclib, and abemaciclib.^34^ The close interplay of the *p16* and *RB* tumor suppressor genes and the activation of CDK is central to the pathophysiology of several NSCLCs.^34^

In our analysis, the frequency of *TP53* inactivation was lower in *MTAP* deficient tumors. The exact clinical implications of the *TP53* alterations in this setting are uncertain. However, studies have shown *TP53* mutated NSCLC to be associated with negative prognosis, especially when combined with mutations in *EGFR* and *ALK*.^35^, ^36^ Our study showed that *MTAP*‐deficient NSCLC had higher genomic alterations in *EGFR*. The NCCN guidelines recommend *EGFR* TKIs for advanced NSCLC with targetable *EGFR* alterations. Contemporary trails like the FLAURA trail have provided evidence for using third‐generation TKIs like osimertinib, especially in NSCLC with brain metastasis.^37^ Given our results, it would be interesting to see the survival and outcome of NSCLC patients with both *EGFR* and *MTAP* alterations and their response to TKIs. Determining whether there would be a survival benefit from combining *PRMT5* or *MAT2A* inhibitors with *EGFR* TKIs merits study. It would also be interesting to see if these novel inhibitors would work in a setting where resistance to anti‐EGFR TKIs emerges in patients with both MTAP loss and EGFR alteration. Figure 2 represents an example of a case of NSCLC, where both *MTAP* loss and a targetable *EGFR* mutation are present. This points to the future clinical development of *MAT2A* and *PRMT5* inhibitors in a combination setting with targeted therapies such as anti‐*EGFR* TKIs. *RB1* mutations, which portend worse outcomes,^38^ were lower, while *SMARCA4* alterations, which may be a marker for susceptibility to CDK4/6 inhibition,^39^ was significantly higher in NSCLC with *MTAP* loss.

In our analysis of *MTAP* deficient NSCLC subjects, while the overall *KRAS* or *STK11* genomic alterations did not show any significant difference, *KRAS* G12C mutations were lower in *MTAP*‐loss versus *MTAP*‐intact tumors. The numbers of subjects with elevated TMB (≥ 10 mut/Mb and ≥ 20 mut/Mb) and positive PD‐L1 were lower compared to *MTAP* intact NSCLC (Table 1). *KRAS* mutated NSCLC has shown to have a higher TMB and hence a better response to checkpoint therapy. The clinical significance *KRAS* mutated NSCLC has shown a better OS when treated with checkpoint therapy compared to the *KRAS* wild type NSCLC. Concurrent *KRAS* and *TP53* alterations have demonstrated higher levels of PD‐L1 positivity and have shown excellent responsiveness to immune checkpoint agents like pembrolizumab. On the other hand, *KRAS* GA when combined with *STK11* GA has been linked to resistance to immune checkpoint agents.^40^ TMB and PD‐L1 status are independent predictors of response to immune checkpoint blockade, with high values indicating better outcomes when checkpoint inhibitors are used.^41^

Despite our large sample size, the lack of clinical information such as past and current treatment and latest clinical outcome status is a limitation of our study. However, our study does highlight the unique genomic makeup of *MTAP* deficient NSCLC and provides a basis from which, further studies assessing therapeutic agents that exploit synthetic lethality‐based approaches in this disease can be designed. Currently, *MTAP*‐loss NSCLC represents a histologically heterogeneous group of lung cancers. *PRMT5* and *MAT2A* inhibitors hold promise for the future treatment of these patients whether they present with or subsequently develop advanced disease and the evaluation in *MTAP* loss in this clinical setting as a route to novel therapies in NSCLC appears warranted.

## Funding information

No external funding source utilized.

## CONFLICT OF INTEREST

All Foundation Medicine co‐authors disclose that they are employees of Foundation Medicine and own shares in Roche Holdings. Authors affiliated with Upstate have no conflict of interest to disclose.

## AUTHOR CONTRIBUTIONS

1. Ashok Kumar, Prashanth: Formal analysis, Validation, Visualization, Roles/Writing ‐ original draft, Writing ‐ review & editing.
2. Graziano, Stephen L: Conceptualization, Data curation, Formal analysis, Investigation, Methodology, Project administration, Resources, Validation, Visualization, Writing ‐ review & editing.
3. Danziger, Natalie: Conceptualization, Data curation, Formal analysis, Funding acquisition, Investigation, Methodology, Project administration, Resources, Software, Visualization, Writing ‐ review & editing.
4. Pavlick, Dean: Conceptualization, Data curation, Formal analysis, Funding acquisition, Investigation, Methodology, Project administration, Resources, Software, Visualization, Writing ‐ review & editing.
5. Severson, Eric A: Conceptualization, Data curation, Formal analysis, Funding acquisition, Investigation, Methodology, Project administration, Resources, Software, Visualization, Writing ‐ review & editing.
6. Ramkissoon, Shakti H: Conceptualization, Data curation, Formal analysis, Funding acquisition, Investigation, Methodology, Project administration, Resources, Software, Visualization, Writing ‐ review & editing.
7. Huang, Richard SP: Conceptualization, Data curation, Formal analysis, Funding acquisition, Investigation, Methodology, Project administration, Resources, Software, Visualization, Writing ‐ review & editing.
8. Decker, Brennan: Conceptualization, Data curation, Formal analysis, Funding acquisition, Investigation, Methodology, Project administration, Resources, Software, Visualization, Writing ‐ review & editing.
9. Ross, Jeffrey S: Conceptualization, Data curation, Formal analysis, Funding acquisition, Investigation, Methodology, Project administration, Resources, Software, Supervision, Validation, Visualization, Roles/Writing ‐ original draft, Writing ‐ review & editing.

## ETHICS STATEMENT

The study was approved by the Western Institutional Review Board (Protocol No. 20152817) which issued an approval waiver of informed consent and a HIPAA waiver of authorization.

## Data Availability

Data is from Foundation Medicine database and archives. Research data are not shared.
